# Effects of the Addition of Crude Fibre Concentrate on Performance, Welfare and Selected Caecal Bacteria of Broilers

**DOI:** 10.3390/ani13243883

**Published:** 2023-12-17

**Authors:** Jakub Urban, Sławomir Jaworski, Agata Lange, Damian Bień, Arkadiusz Matuszewski, Monika Michalczuk

**Affiliations:** 1 Department of Animal Breeding, Institute of Animal Sciences, Warsaw University of Life Sciences, Ciszewskiego 8, 02-786 Warsaw, Poland; damian_bien@sggw.edu.pl (D.B.); monika_michalczuk@sggw.edu.pl (M.M.); 2 Department of Nanobiotechnology, Institute of Biology, Warsaw University of Life Sciences, Ciszewskiego 8, 02-786 Warsaw, Poland; slawomir_jaworski@sggw.edu.pl (S.J.); agata_lange1@sggw.edu.pl (A.L.); 3 Department of Animal Environment Biology, Institute of Animal Sciences, Warsaw University of Life Sciences, Ciszewskiego 8, 02-786 Warsaw, Poland; arkadiusz_matuszewski1@sggw.edu.pl

**Keywords:** crude fibre, welfare, selected caecal bacteria, probiotics

## Abstract

**Simple Summary:**

Incorporating a precise amount of crude fibre additive into animal feed can yield multiple benefits. These include better feed digestibility, decreased litter moisture, and promotion of the probiotic microbiome in the digestive tract. The objective of the study was to evaluate the impact of crude fibre concentrate on key production outcomes, such as final body weight, mortality, and feed conversion ratio, as well as on welfare and the prevalence of certain caecal bacteria (*Enterobacteriaceae* and lactic acid bacteria). Additionally, the study examined the pH of broiler chicken faeces and litter after incorporating crude fibre concentrate into their feed. According to the findings, the incorporation of crude fibre concentrate yielded favourable outcomes on the end body weight, welfare parameter and the number of colony-forming units of lactic acid bacteria within the cecum whilst also resulting in a reduction in the pH of faeces and litter.

**Abstract:**

The study evaluated the effects of crude fibre concentrate supplementation on final body weight, mortality, feed conversion ratio, European Production Efficiency Factor, European Broiler Index, welfare parameters, colony-forming units of selected caecal bacteria (*Enterobacteriaceae* and lactic acid bacteria) and pH of broiler faeces and litter. The study comprised 990 Ross 308 male chicks divided into three groups, a control and two experimental groups, which were given crude fibre concentrate as a feed supplement. On the thirty-fifth day of rearing, the birds’ welfare scores were evaluated, and 2 g of cecum was collected post-mortem from six chickens in each group. Subsequently, a series of ten-fold dilutions of the material was prepared, followed by cultures and measurement of pH in the faeces and litter. The inclusion of crude fibre concentrate resulted in a beneficial impact on the ultimate body mass (*p* ≤ 0.001), welfare standard (*p* ≤ 0.001), and quantity of colony-forming units of lactic acid bacteria (*p* ≤ 0.05) within the cecum. Furthermore, it had a positive influence on lowering the pH levels of both faeces and litter (*p* ≤ 0.05).

## 1. Introduction

Dietary fibre (DF) is an intrinsic element of plant-origin feeds linked to physiological, structural, and functional transformations in the digestive system. DF comprises non-starch polysaccharides (NSP), oligosaccharides, and lignins that resist digestion and enzymatic hydrolysis. Fibre can be classified as soluble or insoluble, depending on its behaviour in water. Both insoluble and soluble fibre are crucial in broiler feed mixtures’ nutritional values. Soluble dietary fibre is believed to enhance intestinal contents’ viscosity, affect the intestinal microbiome’s composition adversely, and reduce nutrient assimilation. However, a group of soluble fibres, which includes oligosaccharides, serves as prebiotics, facilitating the gut microbiota’s development and offering additional benefits. The inclusion of insoluble DF in the diet of broilers has an impact on the gut’s structural morphology, the development of gastrointestinal organs, nutrient absorption, the performance of growth, and gut microbiota [1]. The term ‘crude fibre’ refers to the insoluble dietary fibre fractions. Several definitions exist in the literature about CF. According to Henneberg and Stohmann, CF has been defined in various ways [2]. Crude fibre refers to the organic materials left over when plant structures are dissolved in a weak solution (1.25%) of sulphuric acid and later in a solution of potassium hydroxide (1.25%). According to McDonald and Whitesides [3], crude fibre comprises the constituents of the cell walls found in plant tissues, including lignin, cellulose and hemicellulose. The components of crude fibre are as follows: 50–80% of the total cellulose, 10–15% lignin and only 20% hemicellulose [4,5]. Products with a crude fibre content of at least 60% are referred to as crude fibre concentrates (CFCs). The high fibre content is achieved by using physical or thermomechanical concentration processes. The base component of CFCs is usually lignocellulosic or cellulosic fibre. According to the results of studies presented by many authors [6,7,8,9,10,11,12,13,14], lignin and cellulose have a positive impact on stimulating probiotic bacteria and improving the structure and function of the poultry digestive tract. Incorporating cellulose as a feed additive increases the population of advantageous intestinal microorganisms, reducing the number of harmful ones and thus lowering the risk of poultry pathogens [9,14,15,16]. Advantageous probiotic microorganisms are an integral part of the formation of the gut microbiota, modulate its composition and maintain intestinal homeostasis [17,18,19]. Microbiota is a term that describes the classification of all microorganisms (bacteria, fungi, viruses and archaeons) living in a specific environment (host organism), in this case in the gut. The occurrence of any disturbance in the balance of the gut microbiota is marked by a negative impact on the health and biology of the animal body, as gut integrity, metabolism, nutrition, immunity and the neuroendocrine system all depend on a healthy microbiota [20,21] which is in constant interaction in the microbiota–brain–gut axis [21,22,23]. The balance or homeostasis of a healthy gut is influenced via the previously mentioned microbiota–brain–gut axis, the immune system, oxidative stress, nutrition, the intestinal epithelial barrier, genetic factors and feed additives [24].

A remarkably high water-binding capacity characterises CFCs. Hence, CFCs can efficiently bind water. Water is absorbed in the upper section of the intestine before being released in the lower section under osmotic pressure. This makes the water available for reabsorption and prevents it from appearing in the litter [25]. Litter serves a multitude of vital purposes, such as soaking up and discharging moisture, providing cushioning and insulation against contact with the ground, and facilitating birds’ instinctual activities of burrowing and cleaning themselves [26,27,28]. During the rearing period, litter conditions undergo modifications primarily because of the augmented moisture resulting from the birds’ excreted faecal matter. Heightened litter moisture content diminishes the quality of litter, leading to the provocation of footpad inflammation in broiler chickens [26,27,29]. Furthermore, heightened moisture in the litter encourages the formation of clumps, resulting in a compacted layer on the top of the litter that can range from 5 to 10 cm in thickness [26,30]. The compacted litter surface can retain moisture and become slippery, resulting in adverse effects on bird welfare through heightened incidences of footpad dermatitis and hock burns. Additionally, it increases susceptibility to other diseases [26,27,31,32].

The objective of this investigation was to assess the impact of incorporating crude fibre concentrate into chicken feed for broilers on crucial performance metrics such as final body weight, mortality rate, feed conversion ratio (FCR), European Production Efficiency Factor (EPEF), European Broiler Index (EBI), welfare indicators, and number of colony-forming units for selected caecal bacteria (*Enterobacteriaceae* and lactic acid bacteria), as well as the pH of broiler chicken faeces and litter.

## 2. Materials and Methods

### 2.1. Crude Fibre Concentrate

ARBOCEL^®^ RC fine (J. Rettenmaier & Söhne GmbH + Co KG, Holzmühle 1, 73494 Rosenberg, Germany) lignocellulosic crude fibre concentrate is a type of product with a crude fibre content of 65 g to 70 g per 100 g, and a lignin content of more than 20 g per 100 g, according to the manufacturer’s specifications [14]. The concentrate has a water content of 7.7% and a remarkably high swelling power of 800%. Additionally, it contains crude fibre (65.3%), non-protein nitrogen compounds (25.1%), total protein (1.0%), crude fat (0.3%), and crude ash (0.5%) [33]. ARBOCEL^®^ is derived from newly harvested spruce (*Picea*) trees. It promotes the intestinal villi’s function and boosts the digestive system’s enzymatic activity. Moreover, it is free of mycotoxins and lacks soluble fibres [34].

### 2.2. Animals

The study was conducted on 990 male chicken broilers of the Ross 308 breed, who were assigned randomly to three groups: control (C), experimental 1 (A1), and experimental 2 (A2), with five replications of 66 birds in each. The stocking density on the 42nd rearing day was maintained under 33 kg/m^2^, and the rearing period lasted for 42 days under standard conditions. The chickens were accommodated on floor system with wood pellets, with a light cycle that complied with the Ross Management Guide [35] and with access to fresh water.

The differentiating factor was the addition of crude fibre concentrate to the feed of the experimental groups (Table 1).

During the experimental period, the broilers were fed using the following feeding program: starter, days 1–10; grower I, days 11–21; grower II, days 22–35; and finisher, days 36–42 (Table 2). Before the experiment began, the feed was tested by three independent centres (two Polish and one German) using the near-infrared (NIR) method to determine the chemical composition of the chickens’ diets: the averaged results have been placed in Table 3.

Throughout the experiment, the body weight of the avian subjects was measured on days 1, 10, 21, 35 and 42. To determine the FCR, feed consumption was measured on days 10, 21, 35 and 42. The health of the flock was under constant vigilance by a veterinarian. The EPEF and EBI were calculated using the following prescribed Formulas (1) and (2) [36]:

TWG = body weight (g) at the end − body weight (g) at the start;
ADG (g/chick/d) = TWG/days of growth period;
FCR (kg feed/kg gain) = cumulative feed intake (kg)/total weight gain (kg);
Viability (%) = 100 − Mortality (%)

(1) $$EPEF=\frac{Viability(\%)\times BW(kg)}{Age(d)\times FCR(kg\;feed/kg\;gain)}\times 100$$

(2) $$EBI=\frac{Viability\left(\%\right)\times ADG(g/chick/day)}{FCR(kg\;feed/kg\;gain)\times 10}$$

### 2.3. Welfare Assessment

At 35 days of age, all of the chickens underwent an extensive visual welfare evaluation consisting of assessments for footpad dermatitis, hock burns and plumage cleanliness. The evaluation was performed following the guidelines of the Welfare Quality Assessment Protocol for Poultry [37], and the resulting gait scores were based on the methodology created by Kestin et al. [38]. Any fatalities that occurred during the implementation of the experiment were also meticulously recorded.

### 2.4. Microbiological Analysis

Two grams of chicken cecum were isolated from 35-day-old chicks and incubated in 20 mL of 0.9% NaCl under shaking at 37 °C for 24 h. Next, ten-fold dilutions were prepared up to 10^-8^, and samples were performed in triplicate using the spread plate technique on MRS (De Man–Rogosa–Sharpe agar) (MRS LAB-AGAR™ (BIOMAXIMA, Lublin, Poland), used for the cultivation of lactobacilli. The addition of magnesium, manganese and acetate with the Tween 80 provided an improved medium for growth of lactobacilli) and ENDO (Endo’s Fuchsine Sulphite Infusion Agar) (ENDO LAB-AGAR^TM^ (BIOMAXIMA, Lublin, Poland) where the growth of Gram-positive bacteria is inhibited by sodium sulphite, fuchsin and sodium-lauryl sulphate. The differentiating factors are lactose and basic fuchsin. These factors enable the initial identification of Gram-negative bacteria, based on their ability to ferment lactose) agar plates. This was carried out to determine the numbers of lactic acid bacteria and *Enterobacteriaceae*, respectively. Five grams of the bedding used by the chickens for 35 days were incubated in 50 mL of 0.9% NaCl under shaking conditions at 37 °C for 24 h. Afterwards, ten-fold dilutions were made up to 10^−8^, and then, in triplicate, samples were performed using the spread plate technique on MRS and ENDO agar plates. The results were expressed as CFU (colony-forming units) per mL and transferred onto a log scale.

### 2.5. Measurement of Faeces and Litter pH

On the thirty-fifth day of rearing, 5 g of faeces were collected from nine boxes of birds (3 from each group) along with 5 g of litter from three boxes of each group. These samples were weighed and mixed thoroughly with 20 mL of distilled water. After 15 min, the pH was measured three times for each litter and faeces sample using an ELMETRON CP-401 pH meter equipped with a glass electrode and temperature sensor (Elmetron, Zabrze, Poland).

### 2.6. Statistical Analysis

The statistical analysis of the obtained data was conducted using the computer programs GraphPad and PS IMAGO PRO 8.1, employing one-way ANOVA analysis of variance. To ensure data normality, the Shapiro–Wilk test was employed, while Levene’s test was used to evaluate variance homogeneity. To determine group differences, Tukey’s post hoc test was applied. Only if normality or homogeneity of variance tests provided failure results was the non-parametric Kruskal–Wallis test employed. For group effects, the Kruskal–Wallis test and the Bonferroni method were used for pairwise comparisons.

## 3. Results

### 3.1. Production Performance and Welfare Indicators of Broiler Chickens

On day 42 of rearing, broiler chickens in group A1, who received the experimental feed, had the highest mean body weight among the groups. The A2 group, fed with the experimental mix, had a weight slightly lower than A1. The control group (C) had the lowest final weight, differing from both A1 and A2 (*p* ≤ 0.001) to a greater extent. There were no statistically significant differences in feed consumption (kg/kg body weight gain) based on the mixture used. Group C had the lowest FCR, while group A2 had the highest. Moreover, group C had the highest mortality, whereas group A1 had the lowest. Based on the EPEF and EBI metrics, the broiler chickens in group A1 achieved the highest scores in this experiment, primarily due to their higher final body weight and lower mortality rates than the other groups (as shown in Table 4).

The assessment of footpad dermatitis (FPD) indicated that 58.6% of broiler chickens in group A2 obtained a score of 0, which was the highest percentage (Table 5). On the other hand, in group C, only 42.1% of the birds received a score of 0, the lowest rate. All of the findings achieved statistical significance. In the evaluation of gait score (GS), group A2 broiler chickens (experimental diet) achieved the most satisfactory outcome (score 0). This was due to a more significant proportion of chickens in this group (as many as 84.8%) exhibiting correct movement (*p* ≤ 0.001). Meanwhile, the A1 experimental group had a slightly lower result than the A2 group, with 83.8% of birds receiving a score of 0. Regarding hock burns, the experimental group A2 showed the lowest incidence of initial signs (*p* ≤ 0.001) compared to all other groups. In the control group (C), only 52.1% of chickens exhibited smooth and balanced movements. Chickens showing reluctance to move, difficulty in performing consecutive steps smoothly, or displaying impaired balance were allocated to group C. Regarding plumage cleanliness assessments, experimental group A2 ranked highest with a statistically significant score (*p* ≤ 0.001). In group A1, over 40% of the chickens evaluated received 0 points, indicating optimal plumage cleanliness. The lowest percentage of chickens displaying excellent plumage quality was noted to be in group C, also yielding a significant outcome (*p* ≤ 0.001).

### 3.2. Results of the Microbiological Analyses

#### Results of Microbiological Analyses of Chicken Cecum Contents

**Figure 1:**
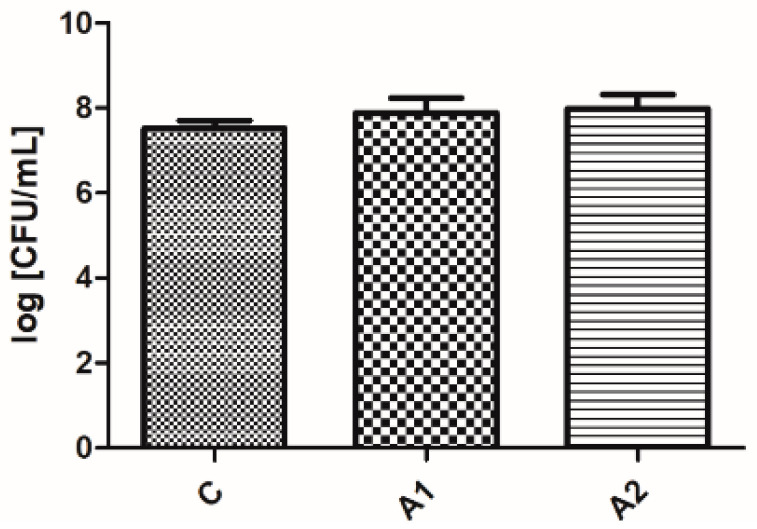
The logarithm of the number of colony-forming units (CFUs) on ENDO medium per mL. C—control group, A1 and A2—groups containing crude fibre concentrate.

**Figure 2:**
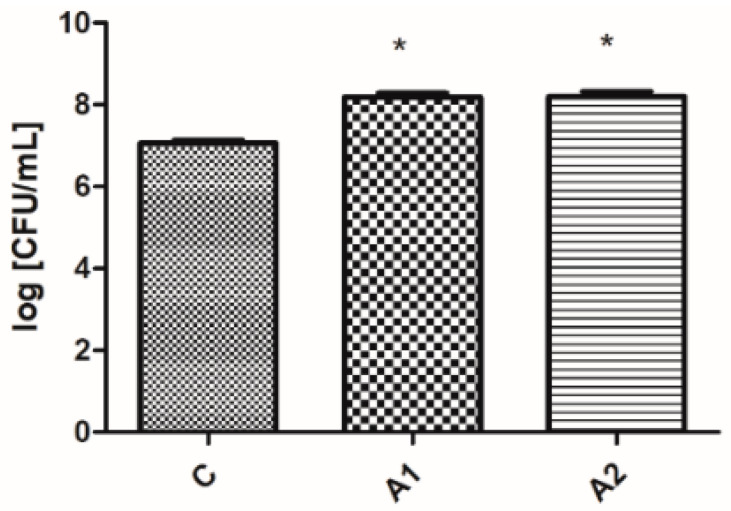
The logarithm of the number of colony-forming units (CFUs) on MRS medium per mL. * means that a column is significantly different at *p* ≤ 0.05. C—control group, A1 and A2—groups containing crude fibre concentrate.

There was no significant difference (*p* ≥ 0.05) in the number of CFUs of *Enterobacteriaceae* observed between the control and experimental groups (refer to Figure 1). For lactic acid bacteria, a higher number of CFUs was observed (*p* ≤ 0.05) in the groups which consumed feed with crude fibre concentrate (refer to Figure 2).

### 3.3. Results of the pH Measurements

#### Results Obtained from Measuring Faeces and Litter pH

**Figure 3:**
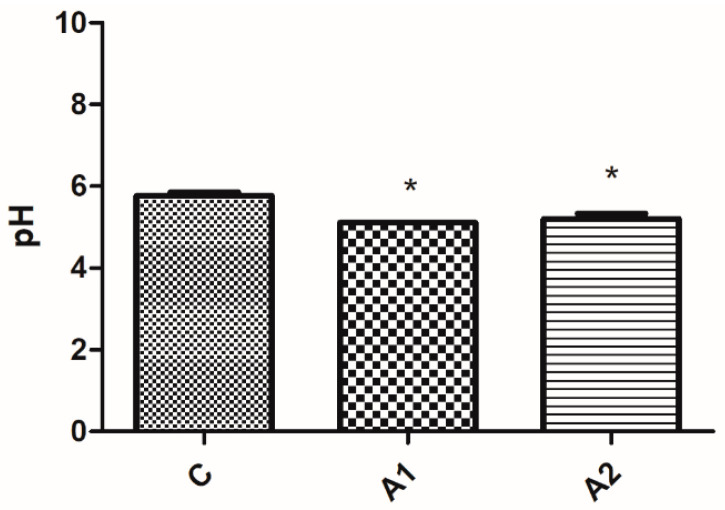
The pH values of the faeces from the birds from individual pens. * means that a column is significantly different at *p* ≤ 0.05. C—control group, A1 and A2—groups containing crude fibre concentrate.

**Figure 4:**
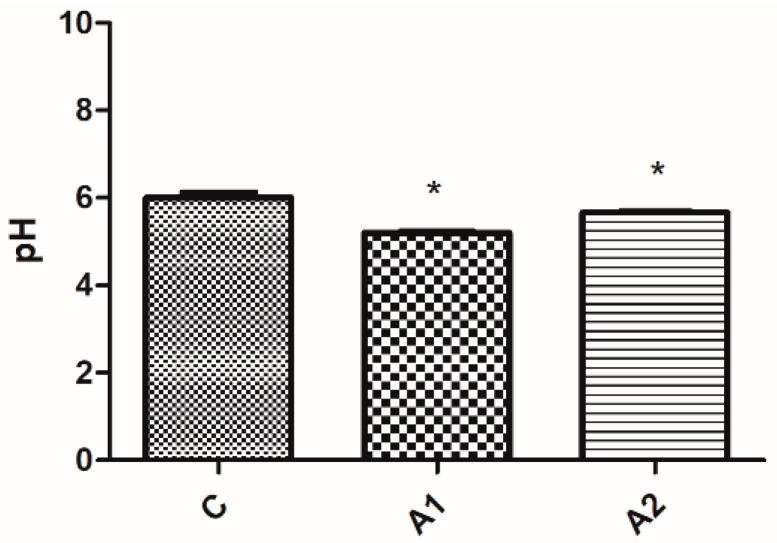
The pH values of the litter from individual pens. * means that a column is significantly different at *p* ≤ 0.05. C—control group, A1 and A2—groups containing crude fibre concentrate.

The pH levels of both faeces and litter were lower (*p* ≤ 0.05) in the experimental groups (refer to Figure 3 and Figure 4).

For both the quantity of lactic acid bacteria colony-forming units and the pH of faeces and litter, there were no significant differences (*p* ≥ 0.05) observed among the experimental groups (A1 and A2) depicted in Figure 2, Figure 3 and Figure 4.

## 4. Discussion

### 4.1. Production Performance of Broiler Chickens

According to Abdollahi et al. [39], the FCR was higher for chicken broilers fed feed mixes containing 1% lignocellulose than the FCR of birds in the control group. In contrast, incorporating lignocellulose at 0.25% to 2% in the feed mixture for chicken broilers from week three to week six decreased the FCR for the experimental groups and increased the birds’ body weight [40,41,42]. In other studies, it has been shown that incorporating a maximum of 0.75% lignocellulose in broiler feed results in reduced FCR and increased body weight gains [40,43,44]. As reported by Sozcu [45], using a processed lignocellulose additive at a rate of 1 kg per tonne of feed had a positive effect on the final body weight and FCR of chicken broilers. Studies available in the literature show that incorporating lignocellulose into chicken broiler feed at a concentration of 2% or less did not adversely impact the production performance of experimental groups [13,43,46,47]. The final production efficiency was also assessed in terms of the EEPEF and EBI [48]. The EPEF is used worldwide as one of the primary indicators of broiler growth performance [49,50,51,52]. In certain countries, EBI serves as an additional indicator to measure the growth performance of broilers and is calculated for flocks at different slaughter ages. It is important to note that the EBI score is always lower than the EPEF score because the average daily weight gain is used to calculate the average EBI [49]. Higher EPEF or EBI index values indicate a more favourable production profit [36,52,53]. In some countries, the EBI operates as an added gauge for determining the growth performance of broilers, calculated for flocks at varying slaughter ages. It is worth noting that the EBI score consistently indicates a lower value than the EPEF score due to the use of average daily weight gain in its computation.

### 4.2. Welfare Indicators of Broiler Chickens

Both hock burns and FPD are important indicators of welfare levels in broiler production [27,54]. These lesions can lead to pathways that allow pathogenic bacteria to enter the body [45,46]; as a result, there are production losses and carcass rejections at the processing plant. In severe cases, these lesions lead to ulceration and inflammation of the subcutaneous tissue, resulting in pain and reduced welfare [54,55]. The moisture content of the litter on which the birds are kept is one factor that influences the rate and severity of footpad inflammation and ankle joint scalds, the decline in locomotor ability, and the cleanliness of plumage. According to Martland [56], wet litter that had a moisture content of 71% caused more cases of contact dermatitis than litter that was drier (58% moisture content) [57]. Wu and Hocking [58] concluded from their study that litter moisture over 30% leads to footpad deterioration [57]. Incorporating lignin-cellulose crude fibre concentrate into the feed lowers the amount of water excreted in faeces and reduces litter humidity due to its water-binding characteristics and ability to “release” water during reflux resorption. This reduction in litter moisture levels contributes positively to the welfare indicators of broiler chickens. According to a study by Professor Farran from the American University of Beirut, adding 0.8% crude fibre concentrate to the feed led to a 10% decrease in litter moisture and eliminated FPD in the experimental group [25]. Verification of footpad dermatitis conducted on the 33rd day of rearing revealed a notably reduced incidence of birds with FPD in the group whose feed was enriched with lignin–cellulose crude fibre concentrate [25]. Adding dietary fibre in the form of lignin–cellulose to chicken broiler feed reduced the incidence of FPD [59].

Feathers protect birds from cold, moisture, and skin infections. Therefore, if feathers become wet or soiled by litter, droppings, or dirt, they may lose their protective properties, which could have a significant impact on bird welfare [37].

### 4.3. Microbiological Analyses and pH Measurements

The investigation of the gut microbiome’s features commenced as early as the 1970s [60,61]. Maintaining a vital equilibrium between advantageous and harmful microbial populations in the gastrointestinal tract can be achieved by promoting the growth of a beneficial microbiome whilst simultaneously reducing the number of pathogenic microorganisms. This approach can result in an enhancement of production performance and an improvement in feed conversion efficiency [45,62].

According to Langenfeld [63], in hens the cecum is where vitamins are synthesised and water is absorbed. In addition, in this part of the digestive tract, the digestion of crude fibre carried out by bacterial enzymes takes place. The digestion of crude fibre is carried out by bacterial enzymes. The polysaccharide fraction of crude fibre under the influence of carbohydrate active enzymes produced by the probiotic microbiota undergoes a process of hydrolysis, resulting in the formation of monomers of carbohydrates [64]. The monomers formed are then used as a substrate in the course of lactic fermentation (carried out by LAB bacteria), resulting in the formation of lactic acid and bacteriocins (protein-structured metabolites characterised by active antimicrobial activity). As a direct result of the production and release of bacteriocins by *Lactobacillus* bacteria, the proliferation of pathogenic bacteria in the intestinal mucosa is inhibited [45,65], making it possible to reduce the size of their numbers in favour of increasing the abundance of probiotic microorganisms in caecal content. Increasing the amount of crude fibre in the diet significantly affects the differences in abundance of different microbial groups in the cecum. According to the available knowledge, it is known that the diet, specifically the proportion of crude fibre in it, modifies the total number of microorganisms [61,66,67]. Based on results from studies conducted to date, it has been shown that feed source and feed modifications can significantly affect the diversity of gut microbial populations, whereas birds fed identical mixed feeds had a strictly stabilised profile of the microbiota [61,68,69]. The increased abundance of *Helicobacter pullorum* and *Megamonas hypermegale* in broilers fed high crude fibre diets implies that these microorganisms play a key role in coordinating the degradation of polysaccharides responsible for the apparent increase in SCFA (Short-Chain Fatty Acids) concentrations [61,70]. An increase in the concentration of added crude fibre in the diet results in an increase in caecal SCFA concentrations. Levels of *Helicobacter pullorum* and *Megamonas hypermegale* are associated with diets that are responsible for higher SCFA production. In contrast, the abundance of the genus *Faecalibacterium* is negatively associated with SCFA production. One of the more interesting observations related to the reduced abundance of bacteria of the genus *Bacteroides* in chickens fed a feed with a low amount of added crude fibre. In addition, it was also shown that an increased amount of added crude fibre concentrate in the feed increased the abundance of *Escherichia coli* and the genus *Campylobacter* in chickens [61]. The state of current knowledge on the modification of the caecal microbiota is consistent with the results of our experiment, where we found that increasing the proportion of crude fibre altered the abundance of the microorganisms we studied and this is related to the previously mentioned mode of fermentation of the polysaccharide fraction of crude fibre in the cecum.

Supplementing broiler feed with 0.25% to 0.6% lignocellulose resulted in a decrease in the population of *Escherichia coli* and *Clostridium perfringens*. Additionally, it led to an increase in the numbers of lactic acid bacteria and *Bifidobacterium* spp. in the ileum and caecum of broiler chickens [42,44]. Similar results were published in a study by Bogusławska-Tryk et al. [13]. The study demonstrated that feed mixtures with 0.25%, 0.5% and 1% lignocellulose led to an increase in the number of *Lactobacillus* spp. in the ileum and the number of *Bifidobacterium* spp. in the ileum and caecum. Furthermore, in the groups receiving feed with 0.25% and 0.5% lignocellulose, the population of *Escherichia coli* and *Clostridium* spp. in the ileum and caecum was reduced [42]. With the inclusion of 0.1% processed lignocellulose in the feed, there was a reduction in the average population size of bacteria from the *Enterobacteriaceae* and *Staphylococcaceae* families alongside a corresponding increase in the population size of *Lactobacillus* spp., a genus responsible for lactic acid production, in the caecal region (*p* ≤ 0.05) [45]. According to a study by Röhe et al. [43], as the proportion of lignocellulose in the feed increased, the presence of *Escherichia, Hafnia*, and *Shigella* in the cecum was reduced by up to 0.5 log10. The reduction was observed in a dose-dependent manner, indicating a potential beneficial effect of lignocellulose. The decrease in pathogenic bacteria populations might stem from the antibacterial characteristics of lignins resulting from the compactness of several phenolic monomers and the abrasive impact of lignocellulose, which diminishes the ability of pathogenic bacteria to bind to the intestinal mucosa’s surface [13,45,71]. Furthermore, lignin has advantageous properties in promoting *Lactobacillus* growth and reducing populations of pathogenic bacteria. According to Bogusławska-Tryk et al. [13], adding 0.5% lignocellulose to the feed substantially boosted lactic acid levels in both the ileum and caecum compared to the control group. According to the findings of the same study, the inclusion of lignocellulose in broiler chicken feed blends, regardless of quantity, resulted in elevated levels of formic acid, acetic acid, propionic acid, and lactic acid in the contents of the ileum and cecum’s digestive systems, as well as butyric acid in the contents of the ileum. An increase in the concentrations of acetic and propionic acid in the digestive contents is desirable as per in vitro studies, which have determined that propionic acid, acetic acid, and formic acid possess toxic effects on certain pathogenic bacteria [13,72,73]. As reported by Van der Wielen et al. [73], there is a noteworthy, robust correlation between increased acetate concentration and decreased numbers of *Enterobacteriaceae* bacteria in the large intestine of broiler chickens [13].

## 5. Conclusions

Adding crude fibre concentrate to the diet of broiler chickens from the experimental groups positively reduced the incidence of welfare parameter disorders such as footpad dermatitis, gait abnormalities, hock burns and plumage cleanliness. Additionally, the inclusion of crude fibre concentrate in the broiler chickens’ feed raised the amount of colony-forming units of selected probiotic microorganisms (from the LAB group) and lowered the pH of the faeces and litter. The addition of crude fibre concentrate led to a considerable augmentation in the ultimate body mass of the birds in both control and experimental groups. Moreover, it enhanced the values of critical parameters like EPEF and EBI.

According to the results obtained from the experiment, the use of a crude fibre concentrate additive is recommended as a positive factor influencing basic production parameters, welfare parameters and the number of probiotic microorganisms in the caecum of broiler chickens.

## Figures and Tables

**Table 1:** Type of feed used and the proportion of fibre added.

Type of Feed	The Proportion of Crude Fibre Concentrate Used (%)		
	C	A1	A2
Starter	0	0.4	0.6
Grower I	0	0.8	1.0
Grower II	0	0.8	1.2
Finisher	0	0.2	0.4

**Table 2:** Formulation of the chickens’ diets.

Ingredient	The Type of Feed			
	Starter	Grower I	Grower II	Finisher
	(g/kg)			
Wheat 13% TP	441.4	479.0	505.2	523.7
Soybean meal	314.5	264.8	233.8	214.0
Maise 8% TP	200.0	200.0	200.0	200.0
Soybean oil	13.43	29.52	36.57	40.66
Limestone	9.32	7.65	7.03	5.68
Monocalcium phosphate	5.05	3.48	2.48	1.40
Premiks *	5.00	5.00	5.00	5.00
Lysine 78.5%	3.46	3.25	3.12	3.05
Methionine 99%	2.98	2.39	2.04	1.81
NaCl	2.26	2.28	2.29	2.29
Sodium carbonate	1.50	1.50	1.50	1.50
L-threonine 98.5%	1.08	1.14	1.02	0.94
**Group**	**Crude fibre concentrate g/kg of feed (on top)**			
C	0.0	0.0	0.0	0.0
A1	4.0	8.0	10.0	2.0
A2	6.0	10.0	12.0	4.0

TP—total protein, NaCl- Sodium Chloride, *—starter 0.5% Sacox, grower 0.5% Sacox (all with the addition of coccidiostats), finisher 0.5% (without coccidiostats).

**Table 3:** Chemical composition of the chickens’ diets.

Component	Group	The Type of Feed			
		Starter	Grower I	Grower II	Finisher
Crude protein (%)	C	20.61	18.78	17.08	18.51
	A1	21.19	18.18	17.00	18.48
	A2	20.76	18.65	16.18	18.30
Crude fat (%)	C	3.06	3.06	2.84	2.87
	A1	3.42	3.02	2.85	2.97
	A2	3.56	3.08	2.70	2.90
Crude fibre (%)	C	3.14	3.20	2.84	3.08
	A1	3.36	3.11	3.35	3.18
	A2	3.27	3.22	3.25	3.37
Ash (%)	C	8.50	5.94	5.11	6.96
	A1	5.35	4.55	5.50	4.38
	A2	5.08	4.67	7.86	4.43

**Table 4:** Results of the chicks’ body weights at day 42 (g), FCR, mortality, EPEF, and EBI.

Indices	Group			SEM	*p* Value
	C	A1	A2		
Body weight, g	2917 ^A^	3140 ^B^	3083 ^B^	10.475	≤0.001
FCR, kg × kg^−1^	1.65	1.68	1.72	0.107	0.355
Mortality, %	4.85	3.34	3.64	0.324	0.126
EPEF, scores	400.5	430.2	411.3	-	-
EBI, scores	395.1	424.8	406.0	-	-

Annotation of the information in the line items: ^A,B^—*p* ≤ 0.01, C—control group, A1 and A2—groups fed crude fibre concentrate; FCR—feed conversion ratio, EPEF—European Production Efficiency Factor, EBI—European Broiler Index.

**Table 5:** Footpad dermatitis, gait score, hock burns, and plumage cleanliness scores at day 35 of the broiler chicken experiment by group.

	Statistic	Score	Group					
			C		A1		A2	
			%	*n*	%	*n*	%	*n*
**Footpad dermatitis**		0	42.1	120	45.7	133	58.6	170
		1	54.7	156	45.4	132	39.3	114
		2	3.2	9	8.9	26	2.1	6
*Kruskal–Wallis test*	*p* ≤ 0.001		A		A		B	
**Gait Score**		0	51.2	146	83.8	244	84.8	246
		1	31.6	90	14.1	41	13.5	39
		2	10.9	31	1.4	4	1.4	4
		3	4.2	12	0.7	2	0.3	1
		4	2.1	6	0.0	0	0.0	0
		5	0.0	0	0.0	0	0.0	0
*Kruskal–Wallis test*	*p* ≤ 0.001		A		B		B	
**Hock Burns**		0	57.9	165	64.6	188	70.0	203
		1	35.1	100	33.3	97	29.7	86
		2	5.6	16	2.1	6	0.3	1
		3	1.4	4	0.0	0	0.0	0
*Kruskal–Wallis test*	*p* ≤ 0.001		A		B		B	
**Plumage cleanliness**		0	53.3	152	90.0	262	90.0	261
		1	44.6	127	10.0	29	10.0	29
		2	2.1	6	0.0	0	0.0	0
*Kruskal–Wallis test*	*p* ≤ 0.001		A		B		B	

Annotation of the information in the line items: ^A,B^—*p* ≤ 0.01; C—control group, A1 and A2—groups fed crude fibre concentrate.

## Data Availability

All data generated or analysed during the study are included in this published article. The datasets used and/or analysed in the current study are available from the corresponding author on reasonable request.
